# Synthesis and Biological Evaluation of Enantiomerically Pure (*R*)- and (*S*)-[^18^F]OF-NB1 for Imaging the GluN2B Subunit-Containing NMDA receptors

**DOI:** 10.21203/rs.3.rs-2516002/v1

**Published:** 2023-01-27

**Authors:** Marvin Korff, Ahmad Chaudhary, Yinlong Li, Xin Zhou, Chunyu Zhao, Jian Rong, Jiahui Chen, Zhiwei Xiao, Nehal H. Elghazawy, Wolfgang Sippl, April T. Davenport, James B. Daunais, Lu Wang, Carmen Abate, Hazem Ahmed, Ron Crowe, Steven H. Liang, Simon M. Ametamey, Bernhard Wünsch, Achi Haider

**Affiliations:** Department of Radiology and Imaging Sciences, Emory University, 1364 Clifton Road, Atlanta, GA 30322, USA.; Department of Radiology and Imaging Sciences, Emory University, 1364 Clifton Road, Atlanta, GA 30322, USA.; Department of Radiology and Imaging Sciences, Emory University, 1364 Clifton Road, Atlanta, GA 30322, USA.; Department of Radiology and Imaging Sciences, Emory University, 1364 Clifton Road, Atlanta, GA 30322, USA.; Department of Radiology and Imaging Sciences, Emory University, 1364 Clifton Road, Atlanta, GA 30322, USA.; Department of Radiology and Imaging Sciences, Emory University, 1364 Clifton Road, Atlanta, GA 30322, USA.; Department of Radiology and Imaging Sciences, Emory University, 1364 Clifton Road, Atlanta, GA 30322, USA.; Department of Radiology and Imaging Sciences, Emory University, 1364 Clifton Road, Atlanta, GA 30322, USA.; Institute of Pharmacy, Department of Medicinal Chemistry, Martin-Luther-University Halle-Wittenberg, W.-Langenbeck-Str. 4, 06120 Halle, Germany.; Institute of Pharmacy, Department of Medicinal Chemistry, Martin-Luther-University Halle-Wittenberg, W.-Langenbeck-Str. 4, 06120 Halle, Germany.; Department of Physiology and Pharmacology, Wake Forest School of Medicine, Winston Salem, NC 27157, USA.; Department of Physiology and Pharmacology, Wake Forest School of Medicine, Winston Salem, NC 27157, USA.; Center of Cyclotron and PET Radiopharmaceuticals, Department of Nuclear Medicine and PET/CT-MRI Center, the First Affiliated Hospital of Jinan University, Guangzhou 510630, China.; Dipartimento di Farmacia-Scienze Del Farmaco, Università Degli Studi di Bari ALDO MORO, Via Orabona 4, Bari 70125, Italy.; Center for Radiopharmaceutical Sciences ETH-PSI-USZ, Institute of Pharmaceutical Sciences ETH, Vladimir-Prelog-Weg 4, 8093 Zurich, Switzerland.; Department of Radiology and Imaging Sciences, Emory University, 1364 Clifton Road, Atlanta, GA 30322, USA.; Department of Radiology and Imaging Sciences, Emory University, 1364 Clifton Road, Atlanta, GA 30322, USA.; Center for Radiopharmaceutical Sciences ETH-PSI-USZ, Institute of Pharmaceutical Sciences ETH, Vladimir-Prelog-Weg 4, 8093 Zurich, Switzerland.; Institut für Pharmazeutische und Medizinische Chemie, Westfälische Wilhelms-Universität Münster, Corrensstraße 48, D-48149 Münster, Germany.; Department of Radiology, Division of Nuclear Medicine and Molecular Imaging Massachusetts General Hospital and Harvard Medical School, 55 Fruit Street, Boston, MA 02114, USA

**Keywords:** N-methyl-D-aspartate (NMDA) receptor, probe development, translational molecular imaging, GluN2B antagonists, positron emission tomography (PET)

## Abstract

GluN2B subunit-containing *N*-methyl-d-aspartate (NMDA) receptors have been implicated in various neurological disorders. Nonetheless, a validated fluorine-18 labeled positron emission tomography (PET) ligand for GluN2B imaging in the living human brain is currently lacking. As part of our PET ligand development program, we have recently reported on the preclinical evaluation of [^18^F]OF-NB1 – a GluN2B PET ligand with promising attributes for potential clinical translation. However, the further development of [^18^F]OF-NB1 is currently precluded by major limitations in the radiolabeling procedure. These limitations include the use of highly corrosive reactants and racemization during the radiosynthesis. As such, the aim of this study was to develop a synthetic approach that allows an enantiomerically pure radiosynthesis of (*R*)-[^18^F]OF-NB1 and (*S*)-[^18^F]OF-NB1, as well as to assess their *in vitro* and *in vivo* performance characteristics for imaging the GluN2B subunit-containing NMDA receptor in rodents. A two-step radiosynthesis involving radiofluorination of the boronic acid pinacol ester, followed by coupling to the 3-benzazepine core structure via reductive amination was employed. The new synthetic approach yielded enantiomerically pure (*R*)-[^18^F]OF-NB1 and (*S*)-[^18^F]OF-NB1, while concurrently circumventing the use of corrosive reactants. *In vitro* autoradiograms with mouse and rat brain sections revealed a higher selectivity of (*R*)-[^18^F]OF-NB1 over (*S*)-[^18^F]OFNB1 for GluN2B-rich brain regions. In concert with these observations, blockade studies with commercially available GluN2B antagonist, CP101606, showed a significant signal reduction, which was more pronounced for (*R*)-[^18^F]OF-NB1 than for (*S*)-[^18^F]OF-NB1. Conversely, blockade experiments with sigma2 ligand, FA10, did not result in a significant reduction of tracer binding for both enantiomers. PET imaging experiments with CD1 mice revealed a higher brain uptake and retention for (*R*)-[^18^F]OF-NB1, as assessed by visual inspection and volumes of distribution from Logan graphical analyses. *In vivo* blocking experiments with sigma2 ligand, FA10, did not result in a significant reduction of the brain signal for both enantiomers, thus corroborating the selectivity over sigma2 receptors. In conclusion, we have developed a novel synthetic approach that is suitable for upscale to human use and allows the enantiomerically pure radiosynthesis of (*R*)-[^18^F]OF-NB1 and (*S*)-[^18^F]OF-NB1. While both enantiomers were selective over sigma2 receptors *in vitro* and *in vivo*, (*R*)-[^18^F]OF-NB1 showed superior GluN2B subunit specificity by *in vitro* autoradiography and higher volumes of distribution in small animal PET studies.

## Introduction

*N*-methyl-d-aspartate (NMDA) receptors are ligand-gated ion channels that belong to the family of ionotropic glutamate receptors (iGluRs). Endowed with a remarkable variety of biological functions, NMDA receptors constitute heterotetrameric complexes composed of combinations of the subunits GluN1, which is processed in eight distinct splice variants, GluN2A-D, and GluN3A-B.^1–4^ Typically, a functional NMDA receptor comprises two glycinebinding GluN1 subunits and at least one glutamate-binding GluN2 subunit. Simultaneous binding of glycine and glutamate initiates NMDA receptor activation, which involves voltagedependent relief of magnesium blockade, depolarization of the postsynaptic membrane and calcium ion influx.^5–7^ While NMDA receptors are key players in neurophysiology, contributing to memory and learning via modulation of synaptic plasticity, the GluN2B subunit-carrying NMDA receptor has been implicated in the pathophysiology of various neurological disorders.^8–16^ Indeed, the role of overstimulation of the excitatory GluN2B subunit in the development of several CNS-related pathologies has been corroborated,^17, 18^ whereas targeting GluN2B-mediated excitotoxicity has been suggested as a promising therapeutic strategy for various diseases, including Alzheimer’s disease (AD), Parkinson’s disease (PD), ischemic stroke, traumatic brain injury, neuropathic pain and depression.^19–30^ Early efforts to develop NMDA receptor antagonists prompted the discovery of NMDA receptor channel blockers such as phencyclidine (PCP), thienylcyclohexylpiperidine (TCP), ketamine, memantine, and MK-801 (dizocilpine). Despite their well-documented therapeutic effi cacy, most of these “broad-spectrum” antagonists were associated with a poor safety profile, potentially owing to the lack of subunit-selectivity.^17, 31, 32^ As such, more recent attempts have focused on the development of GluN2B-selective antagonists, which has become feasible since the discovery of the *N*-terminal domain (NTD) binding site that is located at the interface between GluN1 and GluN2B.^33^ Several GluN2B-selective antagonists have been reported to date – some of which have been advanced to humans, including CP101,606 (traxoprodil)^30^, MK-0657 (CERC-301)^34^ and EVT-101 (NCT01128452). Nonetheless, the development of a suitable GluN2B-selective antagonist for clinical use has proven challenging, at least in part, due to the lack of appropriate non-invasive imaging tools that allow the assessment of target engagement in the human brain.

Positron emission tomography (PET) constitutes a powerful non-invasive molecular imaging modality that allows real-time quantification of biochemical processes.^35^ Accordingly, PET has been established as a reliable tool for CNS-targeted receptor quantification as well as target occupancy studies in preclinical and clinical research.^36^ Given the translational relevance of visualizing drug-receptor interactions to facilitate the development of GluN2B antagonists in the pipeline, strenuous efforts have been devoted to the discovery of a suitable GluN2B PET radioligand in the past two decades. While numerous probes exhibited high *in vitro* specificity and selectivity towards the GluN2B subunit, the vast majority of reported ligands were plagued by unfavourable *in vivo* performance characteristics.^37^ Major drawbacks included low brain penetration, lack of *in vivo* specificity and selectivity, as well as the presence of radiometabolites in the CNS.^37, 38^ We have recently reported on the first successful GluN2B subunit-selective PET radioligand, (*R*)-[^11^C]Me-NB1, that proved to be suitable for visualizing GluN2B *in vitro* and *in vivo*.^39^ The structure of (*R*)-[^11^C]Me-NB1 belongs to a class of 3-benzazepine based ligands, encompassing a series of high-affi nity GluN2B antagonists, that were first reported by Tewes et al.^40^ Of note, (*R*)-[^11^C]Me-NB1 was successfully translated to humans, rendering it the first and only GluN2B-targeted PET radioligand to be clinically validated to date.^41^ Despite the outstanding performance characteristics, the use of (*R*)-[^11^C]Me-NB1 is limited by the short physical half-life of carbon11 (20.3 min), which confines the use of (*R*)-[^11^C]Me-NB1 to facilities with an on-site cyclotron. As part of our efforts to develop a suitable radiofluorinated analog (physical half-life of fluorine-18, 109.8 min), that would allow satellite distribution to hospitals without an on-site cyclotron, we have synthesized and evaluated a series of fluorinated (*R*)-[^11^C]Me-NB1 derivatives^42–49^ – of which [^18^F]OF-NB1 proved to be particularly promising for translation into humans. However, the clinical translation of [^18^F]OF-NB1 requires further optimization of the radiolabeling strategy. In particular, current radiolabeling routes utilize corrosive reactants such as boron tribromide and lead to racemization of enantiomerically pure precursors. However, based on previous observations with this class of compounds^41^, it is anticipated that (*R*)- and (*S*)-[^18^F]OF-NB1 may exhibit distinct enantiomeric behaviors with respect to GluN2B binding specificity and selectivity over sigma receptors. Along this line of reasoning, a synthetic approach that allows the enantiomerically pure synthesis of (*R*)-[^18^F]OF-NB1 and (*S*)-[^18^F]OF-NB1 without racemization is warranted to enable the evaluation of the enantiomers in future human studies. Thus, the aim of this study was to develop a synthesis strategy that is devoid of corrosive reactants and provides enantiomerically pure (*R*)-[^18^F]OF-NB1 and (*S*)-[^18^F]OF-NB1 suitable for human use, as well as to assess their *in vitro* and *in vivo* performance characteristics for imaging the GluN2B subunits of the NMDA receptor in rodents.

## Results And Discussion

While [^18^F]OF-NB1 exhibited outstanding performance characteristics as a GluN2B subunittargeted PET radioligand in preclinical experiments, the original radiosynthesis was plagued by the harsh conditions required to cleave the hydroxyl protecting groups, hampering automatization and clinical translation of the probe (Scheme 1, previous work).^50^ Moreover, when employing enantiomerically pure precursor **1**, racemization of the center of chirality in benzyl position was observed during the reaction with boron tribromide, precluding the synthesis of enantiomerically pure (*R*)- or (*S*)-[^18^F]OF-NB1.^49^ We have now developed a novel synthesis strategy (Scheme 1, this work) that proceeds via a building block approach, thereby opening up several possibilities: (1) precursor **3** for the radiosynthesis does not contain any OH-groups, which alleviated the necessity of protection groups for the radiofluorination. (2) Starting from enantiomerically pure 3-benzazepine building block 5 allowed us to conduct radiolabeling of enantiomerically pure of (*R*)-[^18^F]OF-NB1 and (*S*)-[^18^F]OF-NB1. (3) Additionally, the novel strategy was faster and more effective compared to the linear two-step synthesis, since a chiral HPLC to separate the enantiomers at the end of the radiosynthesis was no longer required. (4) Finally, given the rapid nature of reductive aminations, our proposed building block approach is adoptable to a wide scope of substrates, including those containing functional groups that are not tolerated under nucleophilic radiofluorination conditions.

The concept of building block-based radiochemistry is well established in fluorine-18 chemistry for a variety of reactions^51, 52^, including reductive alkylations with fluorinated benzaldehyde,^53^ however, this is the first successful example of a radiosynthesis that leverages a radiofluorinated aliphatic aldehyde intermediate for subsequent reductive alkylation. The reductive alkylation reaction is chemoselective towards amines over alcohols, as opposed to nucleophilic substitution of an alkyl halide, where overalkylation of tertiary amines and alkylation of alcohols typically occur as side reactions. Boronic acid pinacol ester **3** was used as precursor for the radiosynthesis, as the copper-mediated radiofluorination of arylboronic esters constitutes a reliable and versatile labeling strategy.^54^ Arylboronic esters as precursors can be readily obtained by Miyaura-borylation of aryl halides in one step.^55^ In contrast to other established radio-precursors, oxidative conditions are not required (cf. diaryliodonium salts or iodonium ylides).^56, 57^ Another advantage of copper-mediated radiofluorination of boronic acid esters is that regioselectivity issues during fluorine-18 incorporation are not typically observed, as opposed to the radiofluorination of diaryliodonium salts.^58^

Boronic acid pinacol ester **3** (Scheme 1), which served as precursor for the radiolabeling, was obtained in a multistep synthesis, starting from commercially available 1iodo2halobenzenes **6a–c**. A Heck reaction^59^ with but-3-en-1-ol, followed by isomerization,^60, 61^ gave aldehydes **7a–c** in variable yields and purity (Scheme 2). Indeed, only in the case of 1,2diidodobenzene, the reaction led to pure 4(2iodophenyl)butanal (**7a**). In sharp contrast, the bromo and fluoro analogues, **6b** and **6c**, yielded inseparable mixtures of 4(2halophenyl)butanal (**7b** and **7c**) and undesired regioisomers 3(2haloophenyl)butanal (**8b** and **8c**, 10–11 %, from ^1^H NMR spectra analysis). Thus, despite the low yeld of 11 %, the synthesis was continued with **7a**. Aldehyde **7c** was used as a non-radioactive reference for HPLC method development.

Protection of the aldehyde as an acetal (**9**)^62^ was necessary, as the Miyaura-borylation gave only trace amounts of precursor **3**, when the reaction was performed directly with aldehyde **7a** (Scheme 3). With acetal 9, the borylation^63^ proceeded in 48% yield, followed by hydrolysis^64^ to the pinacol precursor **3** in 91%. With an open-chain diethyl acetal, instead of cyclic ethylene acetal, the hydrolysis of the diethyl acetal required only very mild conditions using catalytic iodine in acetone. Thus, the diethyl acetal **9** ensured the successful Miyaura-borylation and allowed the facile release of the aldehyde functionality, while leaving the boronic ester intact. Accordingly, precursor **3** for the radiosynthesis was synthesized from 1,2diiodobenzene in four steps in an overall yield of %.

The racemic 3-benzazepine building block (*rac*)-**5** was obtained from commercially available 3-benzazepine **11** by cleavage of the benzyl ether via catalytic hydrogenation in 91% yield (Scheme 3). Chiral resolution of 3-benzazepine (*rac*)-**11** or (*rac*)-**5** to obtain enantiomerically pure 3-benzazepines (*R*)-**5** and (*S*)-5 by chiral HPLC was not successful, using both normal and reversed stationary phases and various eluent combinations. Based on previous experience with this class of compounds, chiral resolution was generally possible for tertiary amines bearing a bulky lipophilic substituent.^39^ Therefore, 3-benzazepine **11** was reductively *N*-alkylated with benzaldehyde and NaBH(OAc)_3_ to give the tertiary amine (*rac*)-**12** in 99% yield. It is worthwhile mentioning that the benzyl group was selected to enable simultaneous cleavage of both, the benzyl ether and benzylamine after chiral resolution. Indeed, benzylated 3-benzazepines (*R*)-**12** and (*S*)**12** were successfully separated by chiral HPLC and the enantiomerically pure benzazepine building blocks (*R*)-**5** and (*S*)-**5** were obtained by catalytic hydrogenation of (*R*)-**12** and (*S*)-**12**, respectively.

The radiolabeling was performed using a two-step procedure involving copper-mediated nucleophilic radiofluorination of precursor **3**,^54^ followed by reductive alkylation with the 3-benzazepine building blocks (*rac*)-, (*R*)- and (*S*)-**5**. A summary of radiochemical yields (RCYs), average synthesis time and molar activities of (*rac*)-[^18^F]OF-NB1, (*R*)-[^18^F]OF-NB1 and (*S*)-[^18^F]OF-NB1 is provided in Table 1. It should be noted that attempts to perform the reductive amination prior to the copper-mediated fluorine-18 labeling did not yield the desired product, potentially owing to the interference of the two alcohol groups (**Supporting Information**).

### In vitro autoradiography with rodent brain tissue

**Figure 1:**
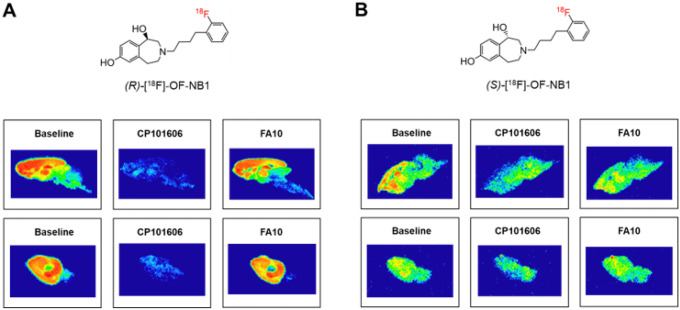
Representative in vitro autoradiography of rat (upper panel) and mouse (lower panel) brains. **A.** Autoradiograms after incubation with (R)-[^18^F]OF-NB1 only (baseline) or in combination with GluN2B antagonist, CP101,606 (10 μM). Selectivity over sigma2 receptors was assessed by blockade studies with FA10 (10 μM). **B.** Autoradiograms after incubation with (S)-[^18^F]OF-NB1 alone or in combination with GluN2B antagonist, CP101,606 (10 μM). FA10 (10 μM) was used to assess selectivity over sigma2 receptors.

GluN2B subunit-specificity and selectivity of (*R*)-[^18^F]OF-NB1 and (*S*)-[^18^F]OF-NB1 were assessed by *in vitro* autoradiography using mouse and rat brain tissue sections. In accordance with reported GluN2B expression patterns in the adult mammalian brain,^65^ a high tracer binding was observed in GluN2B-rich forebrain regions such as the hippocampus, striatum, thalamus and cortex, whereas tracer binding was relatively low in the GluN2B-deficient cerebellum (Fig. 1). Although the latter observations were generally made for both enantiomers, (*R*)-[^18^F]OF-NB1 exhibited a more favorable binding pattern in the rodent brain (Fig. 1A), which was superior to that of (*S*)-[^18^F]OF-NB1 (Fig. 1B) with respect to selectivity for GluN2B-rich brain areas. In concert with these observations, blocking studies with the commercially available GluN2B antagonist, CP101,606 (K_D_ of 10 nM towards GluN2B), revealed a more pronounced reduction of tracer binding for (*R*)-[^18^F]OF-NB1 as compared to (*S*)[^18^F]OF-NB1 on mouse and rat brain sections.

One of the major drawbacks of previously reported GluN2B PET radioligands was off-target binding towards sigma receptors.^39^ While we have previously demonstrated the *in vitro* and *in vivo* selectivity of (*rac*)-[^18^F]OF-NB1 over sigma1 receptors;^44^ it remains unclear whether off-target activity towards sigma2 receptors can also be excluded. As such, we performed additional autoradiography studies by challenging (*R*)-[^18^F]OF-NB1 and (*S*)-[^18^F]OF-NB1 with the previously reported sigma2 ligand, **11b**^66^ (here codenamed FA10, *K*_i_ of 1.4 nM towards sigma2). Overall, no considerable signal reduction was observed for either (*R*)-[^18^F]OF-NB1 or (*S*)-[^18^F]OF-NB1 when employing an excess of FA10 (10 μM), indicating that both enantiomers exhibited selectivity over sigma2 receptors (Fig. 1).

**Figure 2:**
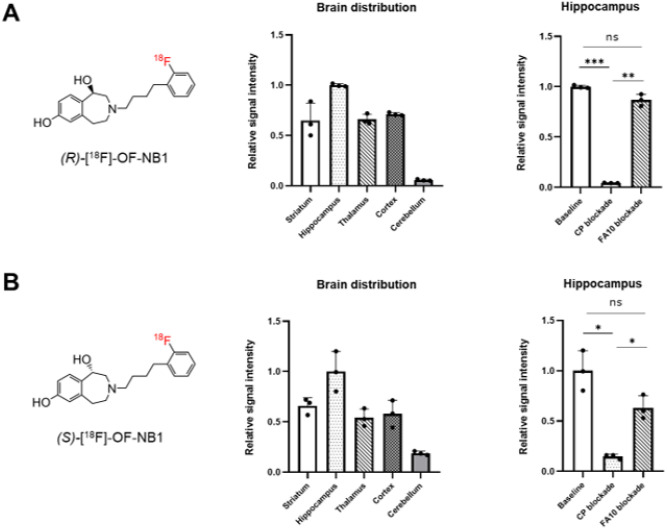
Quantification of autoradiographic data from rat brain sections. **A.** Distribution of (R)-[^18^F]OF-NB1 throughout distinct brain regions. GluN2B subunit-specificity was derived from the extent of signal reduction from baseline to CP (CP101,606) blockade. Selectivity over sigma2 receptors was assessed by the extent of signal reduction from baseline to FA10 blockade. **B.** Distribution of (S)-[^18^F]OF-NB1 throughout distinct brain regions. GluN2B subunit-specificity was derived from the extent of signal reduction from baseline to CP (CP101,606) blockade. Selectivity over sigma2 receptors was assessed by the extent of signal reduction from baseline to FA10 blockade.

Quantification of the autoradiographic data corroborated that highest (*R*)-[^18^F]OF-NB1 binding was observed in the hippocampus, followed by the cortex, striatum and thalamus, whereas the cerebellum showed lowest (*R*)-[^18^F]OF-NB1 binding (Fig. 2A) – with a hippocampus-to-cerebellum ratio of ≈ 20. Although similar quantification patterns were obtained for (*S*)[^18^F]OF-NB1 (Fig. 2B), the hippocampus-to-cerebellum ratio was ≈ 5, indicating that the selectivity for the GluN2B-rich forebrain was less accentuated. While GluN2B blockade studies with CP101606 showed a significant signal reduction, which was more pronounced for (*R*)[^18^F]OF-NB1 (96% signal reduction) than for (*S*)-[^18^F]OF-NB1 (85% signal reduction), sigma2 blockade with FA10 did not reveal a significant signal reduction in the hippocampus for both tracer enantiomers. These results indicated that (*R*)-[^18^F]OF-NB1 exhibits a higher specificity and selectivity for the GluN2B subunit than (*S*)-[^18^F]OF-NB1 *in vitro*.

### PET imaging and in vivo blocking studies

**Figure 3:**
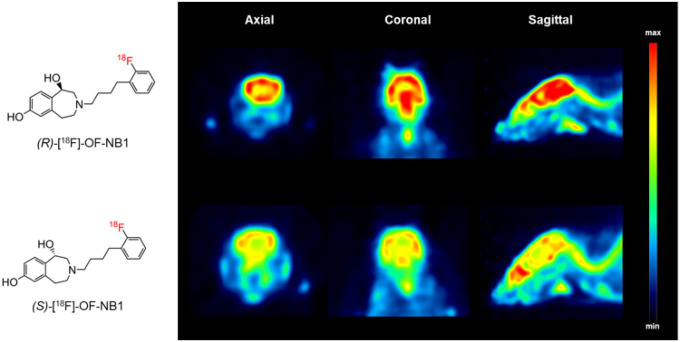
Representative positron emission tomography (PET) images of the mouse brain from an axial, coronal and sagittal view. **A.** Mouse brain images following tail-vein injection of (R)-[^18^F]OF-NB1. **B.** Mouse brain images following tail-vein injection of (S)-[^18^F]OF-NB1.

In a next step, we sought to assess the *in vivo* performance characteristics of (*R*)-[^18^F]OF-NB1 and (*S*)-[^18^F]OF-NB1 by small animal PET imaging. Upon tail-vein injection to CD1 mice, brain uptake was determined by visual inspection of the images as well as by quantification of volumes of distribution (*V*_*T*_) across different brain regions. As depicted in Fig. 3, (*R*)-[^18^F]OF-NB1 exhibited a higher overall uptake in the rodent brain than (*S*)-[^18^F]OF-NB1.

**Figure 4:**
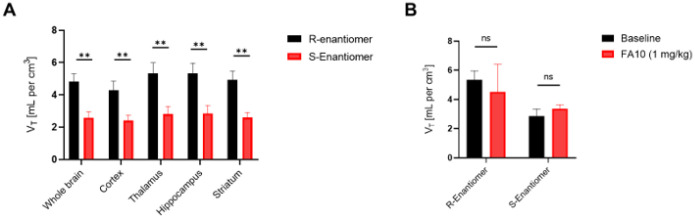
Quantification of (R)-[^18^F]OF-NB1 and (S)-[^18^F]OF-NB1 volumes of distribution (V_T_) across different mouse brain regions, calculated via Logan graphical analysis. **A**. Comparison of (R)-[^18^F]OF-NB1 vs. (S)-[^18^F]OF-NB1 retention in the whole brain, as well as in GluN2B subunitexpressing regions. **B**. Hippocampal volumes of distribution for (R)-[^18^F]OF-NB1 and (S)-[^18^F]OF-NB1 at baseline and following challenge with sigma2 receptor ligand, FA10 (1 mg/kg).

These findings were corroborated by quantification of *V*_*T*_ across different brain regions using Logan plot analyses^67^, where we found significantly higher *V*_*T*_ values for (*R*)-[^18^F]OF-NB1 than for (*S*)-[^18^F]OF-NB1 in the whole brain, as well as in GluN2B-rich brain regions (Fig. 4A). These findings indicated a higher retention of the *R*-enantiomer in the rodent brain. Notable, blockade experiments with sigma2 ligand, FA10 (1 mg/kg), did not result in a significant reduction of the PET signal in the hippocampus (Fig. 4B), or in any other brain region (data not shown), indicating that both enantiomers were selective over sigma2 receptors *in vivo*.

### In vitro autoradiography with non-human primate brain sections

**Figure 5:**
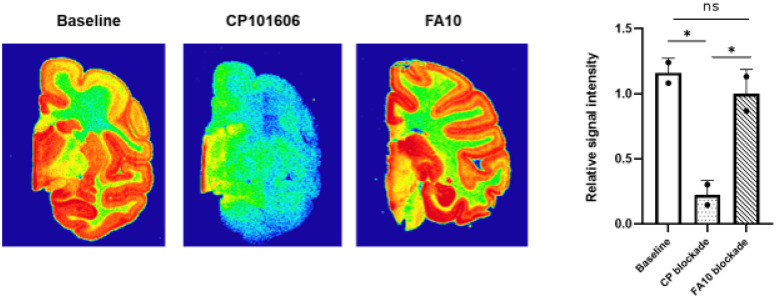
Representative in vitro autoradiograms of (R)-[^18^F]OF-NB1 on post-mortem non-human primate (NHP) brain tissue sections. For specificity testing, GluN2B antagonist, CP101,606, was used in a concentration of 10 μM. Selectivity over sigma2 receptors was assessed using FA10 (10 μM).

Due to the superior performance characteristics of (*R*)-[^18^F]OF-NB1 in rodent studies, we sought to assess its utility for imaging GluN2B subunit-containing NMDA receptors in higher species. As such, post-mortem brain tissue sections from non-human primates (NHPs) were used for autoradiographic testing of (*R*)-[^18^F]OF-NB1. In accordance with observations from rodent studies, we found a heterogenous binding pattern with preferential accumulation of (*R*)-[^18^F]OF-NB1 in GluN2B-rich brain regions (Fig. 5). Employing GluN2B antagonist, CP101,606, a high degree of specificity (80.7% signal reduction) was corroborated. In contrast, blockade studies with FA10 did not reveal a significant reduction of the signal, implying that (*R*)-[^18^F]OF-NB1 is selective over sigma2 receptors in NHPs.

## Conclusion

In the present study, we developed a novel synthetic strategy to obtain enantiomerically pure (*R*)-[^18^F]OF-NB1 and (*S*)-[^18^F]OF-NB1, thereby circumventing the previously reported use of corrosive reagents and allowing for method translation to human-grade production facilities. (*R*)-[^18^F]OF-NB1 significantly outperformed (*S*)-[^18^F]OF-NB1 in autoradiographic experiments using mouse and rat brain, in particular, by exhibiting a higher specificity and selectivity for GluN2B-rich brain regions. While both enantiomers were selective over sigma2 receptors *in vitro* and *in vivo*, small animal PET experiments revealed higher volumes of distribution for (*R*)-[^18^F]OF-NB1 in GluN2B-rich regions of the rodent brain. Overall, these findings suggest that (*R*)-[^18^F]OF-NB1 constitutes a promising GluN2B-targeted PET radioligand for clinical translation.

## Experimental Section

Reagents and solvents were purchased from Sigma-Aldrich, Fisher Scientific, TCI, Combi-Blocks or Labnetwork Inc and were used without further purification. Solvents used in radiosynthesis were purchased in anhydrous grade (puriss., dried over molecular sieves, H_2_O < 0.005%), solvents necessary for extractions, column chromatography and thin-layer chromatography (TLC) were acquired as technical grade. Anhydrous solvents needed for conventional organic synthesis were obtained by drying over molecular sieve (4 Å) under nitrogen atmosphere. Nonaqueous reactions were generally performed under nitrogen atmosphere using flamedried glassware and standard syringe/septa methods. Reactions were magnetically stirred and further monitored by TLC performed on Merck TLC glass sheets (silica gel 60 F_254_). TLC Spots were visualized with UV light (*λ* = 254 nm). Chromatographic purification of products was performed using a Biotage^®^ Selekt System with Biotage^®^ Sfär columns. Reactions at 0°C were carried out using an ice/water bath for cooling purposes. ^1^H and ^13^C NMR spectra were recorded on a Bruker Ascend 600 MHz spectrometer and the chemical shifts (*δ*) are presented in ppm referenced to tetramethylsilane (0 ppm). Analysis of NMR spectra was performed with the MestReNova software (14.2.0). All coupling constants (*J*) are reported in Hz. Multiplicities in the ^1^H NMR spectra are generally given as either s, singlet; d, doublet; dd, doublet of doublets; t, triplet; dt, doublet of triplets; m, multiplet; or bs, broad singlet. High-resolution mass spectrometry (HRMS) was conducted on a ThermoFisher Scientific Exactive^™^ Plus Orbitrap Mass Spectrometer, using atmospheric pressure chemical ionization (APCI) in the positive ionization mode; the resulting data is reported in *m*/*z*. Melting points were determined with a Mel-Temp^®^ capillary melting point apparatus. The radioactivity of samples was quantified with a Capintec CRC^®^-15R radioisotope dose calibrator. Reaction progress in radiochemistry was monitored with Eckert & Ziegler AR-2000 radio-TLC imaging scanner, operated with the WinScan software (3.14). Purification of the radiotracers was performed on a semipreparative HPLC with a Merck Hitachi LaChrome L-7100 pump system, a Shimadzu SPD-10AV UV-vis detector and a Carroll & Ramsey Associates Model 105-S radiation detector, operated with the PowerChrom software (2.7.9). The semipreparative column used was a Venusil MP C18 (10 mm × 250 mm, 5 μm) with an isocratic eluent mixture of water and CH_3_CN (v/v = 65:35, + 0.1% trifluoroacetic acid (TFA)), a flow rate of 5 mL/min for 15 min and UV detection at 254 nm. For quality control, an aliquot of the final formulation of radiotracer was analysed by analytical HPLC, using a LabAlliance Series III pump system, Waters 2487 Dual λ Absorbance Detector UV-vis detector, Carroll & Ramsey Associates Model 105-S radiation detector, operated with the PowerChrom software (2.7.9). The analytical column used was XBridge^™^ Phenyl (4.6 mm × 100 mm, 3.5 μm) with an isocratic mixture of water and CH_3_CN (v/v = 75:25, + 0.1% TFA), a flow rate of 1 mL/min for 25 min (*t*_R_ = 15.9 min) and UV detection at 254 nm. The molar activity of radiotracers was determined by comparing the UV intensity of a sample of the formulated product of known activity, against a calibration curve of the corresponding cold reference of known concentration. The calibration curve was determined from injecting a sample of 80 μL of OF-NB1 solutions in HPLC eluent in six concentrations between 0.1 mg/L and 0.0005 mg/L into the analytical HPLC and measuring the area under the curve of the corresponding UV signal (cf. **Supporting Information**). Animals were purchased from Charles River or Jackson Laboratory and kept under standard conditions. All animal studies were approved and carried out in accordance with the Institutional Animal Care and Use Committee (IACUC) guidelines.

## Chemistry

### General procedure for the synthesis of 4-(2-halophenyl)butanal 7a–7c

A flame dried Schlenk flask was charged with Pd(OAc)_2_ (6 mol%), tetrabutylammonium bromide (1.00 eq.), NaHCO_3_ (2.50 eq.) and molecular sieves (4 Å). The air atmosphere was exchanged by nitrogen in three cycles of evacuation and flushing with N_2_. The reagents were suspended in DMF (dry), the iodohaloaryl compound (**6a–6c**, 1.00 eq.) was dissolved in DMF (dry) and added to the mixture. But-3-en1ol was added and the mixture was stirred at 70°C for 4 h. After cooling down, ethyl acetate was added, and the mixture was filtered through a pad of Celite^®^. It was washed with water and the aqueous layer was extracted with ethyl acetate, the organic layers were combined and concentrated *in vacuo*. This process was repeated two more times. The organic layer was dried with Na_2_SO_4_ and evaporated *in vacuo*. The crude product was purified via flash column chromatography to yield product **7a–c**.

### **4-(2-Iodophenyl)butanal** (**7a**)

Following the general procedure for the synthesis of 4-(2-halophenyl)butanal, Pd(OAc)_2_ (280 mg, 1.25 mmol, 6 mol%), tetrabutylammonium bromide (6.71 g, 20.8 mmol, 1.00 eq.), NaHCO_3_ (4.47 g, 52.0 mmol, 2.50 eq.) and molecular sieves (4 g) were suspended in DMF (60 mL), 1,2diiodobenzene (**6a**, 6.86 g, 20.8 mmol, 1.00 eq.) was dissolved in DMF (15 mL) and added with but-3-en1ol (1.50 g, 20.8 mmol, 1.00 eq.) to the mixture. After cooling down, ethyl acetate (100 mL) was added and after filtration it was washed with water (300 mL) and the aqueous layer was extracted with ethyl acetate (3 × 100 mL). The residue was purified via flash column chromatography (hexanes/ethyl acetate = 1:0 → 95:5). Yellow oil, yield 639 mg (2.33 mmol,11 %). TLC: 0.39 (hexanes/ethyl acetate = 9:1). ^1^H NMR (600 MHz, DMSO-*d*_*6*_): *δ* (ppm) = 1.81 (quint d, *J* = 7.6/1.6 Hz, 2H, C*H*_*2*_CH_2_CHO), 2.47–2.54 (m, 2H, C*H*_*2*_CHO), 2.68 (t, *J* = 7.6 Hz, 2H, C*H*_*2*_CH_2_CH_2_CHO), 6.97 (td, *J* = 7.6/1.8 Hz, 1H, 5-H_iodophenyl_), 7.31 (d, *J* = 7.7 Hz, 1H, 3-H_iodophenyl_), 7.35 (t, *J* = 7.2 Hz, 1H, 4-H_iodophenyl_), 7.83 (d, *J* = 7.9 Hz, 1H, 6-H_iodophenyl_), 9.67–9.72 (m, 1H, CHO). ^13^C NMR (151 MHz, DMSO-*d*_*6*_): *δ* (ppm) = 22.3 (1C,*C*H_2_CH_2_CHO), 39.1 (1C, *C*H_2_CH_2_CH_2_CHO), 42.3 (1C, *C*H_2_CHO), 100.7 (1C, C-1iodophenyl), 128.3 (1C, C-5iodophenyl), 128.6 (1C, C4iodophenyl), 129.7 (1C, C-3iodophenyl), 139.1 (1C, C-6_iodophenyl_), 143.9 (1C, C-2_iodophenyl_), 203.1 (1C, CHO). HRMS: *m*/*z* = 256.9820, calcd. 256.9822 for C_10_H_10_I^+^ [M + H-H_2_O]^+^.

### **4-(2-Bromophenyl)butanal (7b) / 3-(2bromophenyl)butanal** (**8b**)

Following the general procedure for the synthesis of 4-(2-halophenyl)butanal, Pd(OAc)_2_ (40.4 mg, 180 μmol, 6 mol%), tetrabutylammonium bromide (967 mg, 3.00 mmol, 1.00 eq.), NaHCO_3_ (630 mg, 7.50 mmol, 2.50 eq.) and molecular sieves (600 mg) were suspended in DMF (10 mL), 1iodo-2-bromobenzene (**6b**, 849 mg, 3.00 mmol, 1.00 eq.) was dissolved in DMF (2 mL) and added with but-3-en1ol (325 mg, 4.5 mmol, 1.50 eq.) to the mixture. After cooling down, ethyl acetate (10 mL) was added, after filtration it was washed with water (50 mL) and the aqueous layer was extracted with ethyl acetate (3 × 50 mL). The crude product was purified via flash column chromatography (hexanes/ethyl acetate = 1:0 → 9:1) and yielded the product **7b** as a yellow oil (521 mg, 2.29 mmol, 6 %), containing about 0 % of undesired regioisomer 3-(2bromophenyl)butanal (**8b**). TLC: 0.40 (hexanes/ethyl acetate = 9:1). ^1^H NMR (600 MHz, DMSO*d*_*6*_): *δ* (ppm) = 1.19* (d, *J* = 6.2 Hz, 0.3H, CH_3_), 1.77–1.88 (m, 2H, CH_2_), 2.46–2.52 (m, 1.8H, CH_2_), 2.65–2.72 (m, 1.8H, CH_2_), 2.76–2.86* (m, 0.2H, CH_2_), 3.69* (h, *J* = 7.0 Hz, 0.1H, C*H*CH_3_), 7.13–7.17 (m, 0.9H, 5-H_bromophenyl_), 7.30–7.36 (m, 1.8H, 3/4-H_bromophenyl_), 7.56–7.60 (m, 0.9H, 6-H_bromophenyl_), 9.63–9.64* (m, 0.1H, CHO), 9.67–9.68 (m, 0.9H, CHO). * indicates observed isolated proton signals of regioisomer 3(2bromophenyl)butanal in relative intensity of 10%. HRMS: *m*/*z* = 208.9962, calcd. 208.9960 for C_10_H_10_^79^Br^+^ [M + H-H_2_O]^+^.

### **4-(2-Fluoroophenyl)butanal (7c) / 3-(2fluorophenyl)butanal** (**8c**)

Following the general procedure for the synthesis 4-(2-halophenyl)butanal, Pd(OAc)_2_ (40.4 mg, 180 μmol, 6 mol%), tetrabutylammonium bromide (967 mg, 3.00 mmol, 1.00 eq.), NaHCO_3_ (630 mg, 7.50 mmol, 2.50 eq.) and molecular sieves (600 mg) were suspended in DMF (10 mL), 1iodo-2-fluorobenzene (**6c**, 666 mg, 3.00 mmol, 1.00 eq.) was dissolved in DMF (2 mL) and added with but-3-en1ol (324 mg, 4.5 mmol, 1.50 eq.) to the mixture. After cooling down, ethyl acetate (10 mL) was added, after filtration it was washed with water (50 mL) and the aqueous layer was extracted with ethyl acetate (3 × 50 mL). The crude product was purified via flash column chromatography (hexanes/ethyl acetate = 1:0 → 9:1) and yielded the product **7c** as a yellow oil (351 mg, 2.11 mmol, 0 %), containing about 1 % of the undesired regioisomer 3-(2-fluorophenyl)butanal (**8c**). TLC: 0.43 (hexanes/ethyl acetate = 9:1). ^1^H NMR (600 MHz, DMSO-*d*_*6*_): *δ* (ppm) = 1.21* (d, *J* = 7.0 Hz, 0.33H, CH_3_), 1.81 (p, *J* = 7.6 Hz, 1.78H, CH_2_), 2.46 (td; *J* = 7.3, 1.5 Hz; 1.78H; CH_2_), 2.61 (t, *J* = 7.7 Hz, 1.78H, CH_2_), 2.72–2.85* (m, 0.22H, CH_2_), 3.57* (h, *J* = 7.1 Hz, 0.11H, C*H*CH_3_), 7.10–7.16 (m, 1.78H, aryl), 7.22–7.27 (m, 0.89H, aryl), 7.29 (td; *J* = 7.7, 1.8 Hz; 0.89H; aryl), 7.35* (td; *J* = 7.9, 1.9 Hz; 0.11H; aryl), 9.62* (t, *J* = 1.7 Hz, 0.11H, CHO), 9.66 (t, *J* = 1.5 Hz, 0.89H, CHO). * indicates isolated proton signals of regioisomer 3(2fluorophenyl)butanal in relative intensity of 11%. HRMS: *m*/*z* = 149.0762., calcd. 149.0761for C_10_H_10_F^+^ [M + H-H_2_O]^+^.

### 4-(2-Iodophenyl)butanal diethyl acetal (9)

4-(2-iodophenyl)butanal (**7a**, 600 mg, 2.19 mmol, 1.00 eq.), ethanol (510 μL, 8.76 mmol, 4.00 eq.) and *p*-toluenesulfonic acid monohydrate (83 mg, 0.44 mmol, 0.20 eq.) were dissolved in hexanes at 0°C. Molecular sieves (400 mg) were added and the mixture was stirred at 0°C. After 30 min, a second portion of *p*-toluenesulfonic acid monohydrate (83 mg, 0.44 mmol, 0.20 eq.) was added, together with additional molecular sieves (200 mg) and the mixture was stirred for further 20 min at 0°C. The mixture was filtered over a pad of cotton, which was extracted with hexanes and ethyl acetate (10 mL each). The solvent was evaporate*d in vacuo* and the residue was purified via flash column chromatography (hexanes/ethyl acetate = 1:0 → 95:5). and yielded the product **9** as a yellow oil (591 mg, 1.70 mmol, 78%), which was directly used for the subsequent reaction. TLC: 0.50 (hexanes/ethyl acetate = 95:5).

### **4-[2-(4,4,5,5-Tetramethyl-1,3,2-dioxaborolan-2yl)phenyl]butanal diethyl acetal (10**)

A flame dried Schlenk flask was charged with bis(pinacolato)diboron (802 mg, 3.16 mmol, 2.00 eq.), Pd(dppf)Cl_2_ (69.3 mg, 94.8 μmol, 6 mol%) and KOAc (543 mg, 5.53 mmol, 3.50 eq.). The air atmosphere was exchanged by N_2_ in three cycles of evacuation and flushing with nitrogen. After suspending the mixture in DMF (15 mL, dry) and adding iodoacetal 9 (550 mg, 1.58 mmol, 1.00 eq.) dissolved in DMF (5 mL, dry), the reaction mixture was stirred at 80°C for 4 h. After cooling down, the mixture was diluted with ethyl acetate (30 mL) and filtered over a pad of Celite^®^. The mixture was concentrated *in vacuo*, diluted with H_2_O (100 mL) and extracted with ethyl acetate (3 × 50 mL). The organic layers were combined and concentrated *in vacuo* and the extraction procedure was repeated two more times. The combined organic layers were dried over Na_2_SO_4_ and the solvent was removed under reduced pressure. The residue was purified via flash column chromatography (hexanes/ethyl acetate = 1:0 → 95:5) and yielded the product 10 as a yellow oil (264 mg, 758 μmol, 48%), which was directly used for the subsequent reaction. TLC: 0.48 (hexanes/ethyl acetate = 9:1).

### 4-(2-(4,4,5,5-Tetramethyl-1,3,2-dioxaborolan-2-yl)phenyl)butanal (3)

Diethyl acetal **10** (240 mg, 693 μmol, 1.00 eq.) and iodine (18 mg, 69 μmol, 0.10 eq.) were dissolved in acetone (2.5 mL) which was prior dried over Na_2_SO_4_. The reaction mixture was stirred at rt for 40 min. The mixture was diluted with ethyl acetate (20 mL) and washed with half concentrated aqueous Na_2_SO_3_ solution (20 mL). The aqueous layer was extracted with ethyl acetate (20 mL) and the combined organic layers were dried over Na_2_SO_4_ and the solvent was removed *in vacuo*. The residue was purified via flash column chromatography (hexanes/ethyl acetate = 97:3 → 94:6). Yellow oil, yield 172 mg (628 μmol, 91%). TLC: 0.38 (hexanes/ethyl acetate = 9:1). ^1^H NMR (600 MHz, CDCl_3_): *δ* = 1.34 (s; 12H; CH_3_), 1.92 (p; *J* = 7.4 Hz; 2H; C*H*_*2*_CH_2_CHO), 2.44 (td; *J* = 7.3, 2.0 Hz; 2H; C*H*_*2*_CHO), 2.89–2.97 (m; 2H; C*H*_*2*_CH_2_CH_2_CHO), 7.17 (d; *J* = 7.7 Hz; 1H; 6-H_phenylalkyl_), 7.20 (td; *J* = 7.4, 1.2 Hz; 1H; 4-H_phenylalkyl_), 7.36 (td; *J* = 7.5, 1.6 Hz; 1H; 5-H_phenylalkyl_), 7.80 (dd; *J* = 7.4, 1.6 Hz; 1H; 3-H_phenylalkyl_), 9.77 (t; *J* = 2.0 Hz; 1H; CHO). ^13^C NMR (151 MHz, CDCl_3_): *δ* = 25.0 (4C, CH_3_), 25.7 (1C; *C*H_2_CH_2_CHO), 35.1 (1C; *C*H_2_CH_2_CH_2_CHO), 43.6 (1C; *C*H_2_CHO), 83.7 (2C; *C*(CH3)2), 125.5 (1C; C4_phenylalkyl_), 129.5 (1C; C-6_phenylalkyl_), 131.2 (1C; C5_phenylalkyl_), 136.5 (1C; C-3_phenylalkyl_), 148.6 (1C, C-1_phenylalkyl_), 203.2 (1C, CHO), C-2_phenylalkyl_ was not observed. HRMS: *m*/*z* = 275.1813., calcd. 275.1813 for C_16_H_24_O_3_B^+^ [M + H]^+^.

#### (rac)-2,3,4,5-Tetrahydro-1 H -3-benzazepine-1,7-diol ((rac)-5)

A flask was charged with 3-benzazepine **11** (500 mg, 1.86 mmol, 1.00 eq.) and Pd/C (200 mg, 10 wt%) and THF (25 mL) was added. The air atmosphere was exchanged with a hydrogen atmosphere, by flushing the flask with H_2_ for 10 min. A balloon with H_2_ was connected to the flask and the reaction mixture was stirred at 60°C overnight. After cooling down, the mixture was filtered over Celite^®^ and the filter was extracted with MeOH (6 × 30 mL). The solvent was removed *in vacuo*. Beige solid, mp 173°C (decomposition), yield 303 mg (1.69 mmol, 91%). ^1^H NMR (600 MHz, DMSO-*d*_*6*_): *δ* (ppm) = 2.56–2.68 (m, 3H, 2-H, 4-H, 5-H), 2.75–2.81 (m, 1H, 4-H), 2.82–2.88 (m, 2H, 2-H, 5-H), 4.48 (d, *J* = 7.5 Hz, 1H, 1-H), 5.08 (bs, 1H, CHO*H*), 6.46 (d; *J* = 2.5 Hz; 1H, 2-H), 6.49 (dd, *J* = 8.1/2.5 Hz, 1H, 6-H), 7.11 (d, *J* = 8.2 Hz, 1H; 5-H), 9.13 (bs, 1H, PhOH). A signal for the NH proton is not observed in the spectrum. ^13^C NMR (151 MHz, DMSO-*d*_*6*_): *δ* (ppm) = 39.6 (1C, C-5), 48.1 (1C, C-4), 55.5 (1C, C-2), 73.7 (1C, C-1), 111.5 (1C, C-6), 116.5 (1C, C-2), 127.2 (1C, C-5), 135.4 (1C, C-4), 141.3 (1C, C-3), 155.6 (1C, C-1). HRMS: *m*/*z* = 162.0914., calcd. 162.0913 for C_10_H_12_NO^+^ [m + H-H_2_O]^+^.

#### (rac)-3-Benzyl-7-(benzyloxy)-2,3,4,5-tetrahydro-1 H -3-benzazepin-1-ol ((rac)-12)

3-Benzazepine (*rac*)-11 (100 mg, 371 μmol, 1.00 eq.) and benzaldehyde (45 μL, 0.45 mmol, 1.2 eq.) were suspended in THF (5 mL, dry). Under stirring, NaBH(OAc)_3_ (197 mg, 928 μmol, 2.50 eq.) was added at once and the mixture was stirred at rt overnight. An aqueous saturated NH_4_Cl solution (5 mL) and water (15 mL) were added and the mixture was extracted with ethyl acetate (3 × 20 mL). The combined organic layers were dried (Na_2_SO_4_) and the solvent was removed under reduced pressure. The residue was purified via flash column chromatography (CH_2_Cl_2_/CH_3_OH (+ 1% conc. NH_3 aq_.) = 99:1 → 90:10). Yellow viscous oil, yield 133 mg (370 μmol, 99%). ^1^H NMR (600 MHz, CDCl_3_): *δ* (ppm) = 2.64–2.67 (m, 1H, 4-H), 2.78–2.83 (m, 2H, 5-H, 2-H), 3.10–3.13 (m, 1H, 4-H), 3.32–3.39 (m, 2H, 2-H, 5-H), 3.95 (s, 2H, PhC*H*_*2*_N), 4.84 (d, *J* = 7.0 Hz, 1H, 1-H), 5.02 (s, 2H, PhC*H*_*2*_O), 6.70 (d, *J* = 2.6 Hz, 1H; 2-H_phenoxy_), 6.75 (dd, *J* = 8.3/2.7 Hz, 1H, 6-H_phenoxy_), 7.17 (d, *J* = 8.3 Hz, 1H, 5-H_phenoxy_), 7.29–7.43 (m, 10H, H_benzyl_). ^13^C NMR (151 MHz, CDCl_3_): *δ* (ppm) = 34.9 (1C, C-5), 55.1 (1C, C-4), 60.2 (C-2), 63.6 (1C, PhCH_2_N), 70.1 (1C, Ph*C*H_2_O), 71.3 (1C, C-1), 111.7 (1C, C6_phenoxy_), 117.3 (1C, C-2_phenoxy_), 127.6 (2C, C-2/6_benzoxyl_), 128.1 (1C, C4_benzoxy/benzaminyl_), 128.6 (1C, C-4_benzoxyl/benzaminyl_), 128.7 (2C, C-3/5_benzoxyl/benzaminyl_), 128.9 (2C, C-3/5_benzoxyl/benzaminyl_), 129.4 (1C, C-5_phenoxy_), 130.2 (2C, C-2/6_benzaminyl_), 134.6 (1C, C4_phenoxy_/C-1_benzaminyl_), 134.9 (1C, C-4_phenoxy_/C-1_benzaminyl_), 137.0 (1C, C1_benzoxyl_), 140.3 (1C, C3_phenoxy_), 158.4 (1C, C-1_phenoxy_). HRMS: *m*/*z* = 360.1955., calcd. 360.1958 for C_24_H_26_NO_2_^+^ [m + H]^+^.

#### Separation of (R)- and (S)-3-benzyl-7-(benzyloxy)-2,3,4,5-tetrahydro-1 H -3-benzazepin-1-ol ((R)-/(S)-12)

Enantiomeric purification of (*rac*)-**12** was performed by semi-preparative HPLC with an Agilent 1260 Inifinity II system, operated with the OpenLab ChemStation software, and a normal phase ReproSil Chiral-NR (8 μm, 250mm × 10 mm) column using an isocratic eluent mixture of hexanes and isopropanol (v/v = 8:2), with a flowrate of 5 mL/min for 40 min and UV detection at 210 and 250 nm. Racemic 12 (80 mg) was dissolved in a mixture of hexanes and isopropanol (15 mL, v/v = 2:1). The separation was performed in three injections with each 5 mL of this solution. The (*S*)-enantiomer eluted first with a retention time of 9.1 min and was isolated as colorless oil, yield 36.2 mg (91%). The (*R*)-enantiomer eluted second with a retention time of 24.5 min and was isolated as viscous colorless oil, yield 39.8 mg (99%). Enantiomeric purity was determined by injecting a sample of separated (*R*)-**12** and (*S*)-**12**, respectively into the HPLC system. The corresponding chromatograms are shown in the **Supporting Information**.

#### (R)-2,3,4,5-Tetrahydro-1 H -3-benzazepine-1,7-diol ((R)-5)

(*R*)-**12** (39.8 mg, 111 μmol) was dissolved in THF (5 mL, dry) and Pd/C (10 mg, 10 wt%) was added. The air atmosphere was exchanged with a hydrogen atmosphere, by flushing the flask with H_2_ for 10 min. A balloon with H_2_ was connected to the flask and the reaction mixture was stirred at 60°C overnight. After cooling down, the solvent was removed *in vacuo* and the residue was suspended in CH_3_OH (10 mL) and passed through a syringe filter. The solvent was removed under reduced pressure and the residue was suspended in ethyl acetate (5 mL) and passed through a pad of cotton and washed with ethyl acetate. The residue on the filter was dissolved in CH_3_OH and passed through the filter. The solvent was removed *in vacuo*. Colorless solid, yield 9.7 mg (54 μmol, 49%). The analytical data are in agreement with the data of (*rac*)-**5**.

#### (S)-2,3,4,5-Tetrahydro-1 H -3-benzazepine-1,7-diol ((S)-5)

(*S*)-X (36.2 mg, 101 μmol) was reacted and worked-up as described above for (*R*)-X. Colorless solid, yield 11.7 mg (65.3 μmol, 65%). The analytical data are in agreement with (*rac*)-**5**.

## Radiochemistry

[^18^F]Fluoride ions were produced by bombardment of 98% enriched [^18^O]H_2_O via the ^18^O(*p*,*n*)^18^F nuclear reaction. Aqueous [^18^F]fluoride was trapped on a preconditioned anionexchange cartridge (Waters Sep-Pak^®^ Plus Light QMA, preconditioned with 10 mL sat. NaHCO_3_ aq. and 10 mL water).

For the building block approach, [^18^F]fluoride was eluted with a solution of tetraethylammonium bicarbonate (TEAB) in methanol (1.5 mL, 1 mg/mL) into a reaction vial, followed by azeotropic drying with CH_3_CN (1 × 1 mL). Boronic ester **3** (2 mg) and Cu(OTf)_2_(py)_4_ (6 mg) were dissolved in 300 μL of a mixture of dry *N*,*N*dimethylacetamide (DMA) and dry *n*-BuOH (v/v = 2:1) and added into the reaction vial. The mixture reacted in the open vial at 110°C for 20 min. After cooling down, the mixture was diluted with water (8 mL) and passed through a C18-catridge (Waters Sep-Pak^®^ Plus Light C18, preconditioned with 5 mL EtOH and 5 mL water), the cartridge was washed with water (5 mL) and thoroughly dried in air stream. The organic mixture trapped on the cartridge was eluted with dry DMF (0.5 mL) into a reaction vial charged with 3-benzazepine **5** (3 mg), NaBH(OAc)_3_ (4.5 mg) and a stir bar, and the mixture was stirred at 60°C for 30 min. After cooling down, the mixture was diluted with 4 mL of a mixture of water/CH_3_CN (v/v = 65:35, + 0.1% TFA) and purified via semipreparative HPLC (*t*_R_ = 10.0 min). The collected fractions were diluted with 30 mL of water and passed through an a C18 cartridge (Waters Sep-Pak^®^ Plus Light C18, preconditioned with 5 mL EtOH and 5 mL water) and the cartridge was washed with water (5 mL). [^18^F]OF-NB1 was eluted with EtOH (0.6 mL) from the cartridge into the final formulation vial. Radiochemical yields, average reaction times and molar activities are shown above in Table 1. Conditions for the single step radiosynthesis approach can be found in the **Supporting Information**.

### In vitro autoradiography

In vitro autoradiography studies were performed as previously described, however, with minor modifications. ^4^2 Tissue-TEK (O.C.T.) was utilized to embed rodent and NHP postmortem brain tissue, which was subsequently prepared as 20 μm thick tissue sections on a cryostat and mounted on glass slides. The slides were then stored at −80°C until the time of utilization. Brain sections were initially thawed for 10 min on ice prior to *in vitro* autoradiography experiments. They were then preconditioned for 10 min in the assay buffer (pH 7.4) composed of 30 mM HEPES, 0.56 mM MgCl_2_, 110 mM NaCl, 5 mM KCl, 3.3 mM CaCl_2_ and 1% fatty acidfree bovine serum albumin (BSA) at ambient temperature. The tissue sections were then dried and subsequently incubated for 30 min at room temperature with (*R*)- or (*S*)-[^18^F]OF-NB1 solution, respectively. For blockade conditions, 10 μM of the respective blocker were added. These blockers included CP101606 (GluN2B ligand) and FA10 (sigma2 ligand). After incubation, the brain sections were washed in assay buffer for 5 minutes followed by washing buffer (same as assay buffer but without BSA) for 2 × 2 min. They were then dipped twice in distilled water for 5 seconds, subsequently dried and exposed to a phosphor imager plate for 180 min. The plates were scanned and on an Amersham Typhoon scanner, whereas ImageQuant TL 8.1 and ImageJ v1.53e were utilized for image analyses.

## PET imaging

PET imaging studies were performed under Institutional Animal Care and Use Committee (IACUC) guidelines. Female CD-1 mice (10–12 weeks of age) were kept under a 12-h light/12-h dark cycle, with ad libitum access to food and water. On the day of experiment, mice were scanned using a G8 PET scanner (Sofie) under 1–2% isoflurane in air/oxygen 1:1 anesthesia. Body temperature was monitored and maintained by a heating pad installed in the scanner bed. The tracer solution containing 0.6–1.5 MBq (*R*)- or (*S*)[^18^F]OF-NB1 in 1 % ethanol and PBS (100–150 μL per mouse) was injected by use of preinstalled tail-vein catheter. Dynamic PET images were recorded for 60 minutes. For blocking experiments, intravenous injection of the sigma2-selective ligand, FA10 (1 mg/kg), dissolved in 1 % ethanol in PBS (50–100 μL), was performed shortly before tracer injection. PMOD (Zurich, Switzerland) software was used for the reconstruction of the dynamic PET images and volumes of interest were defined as previously described.^68^ Volumes of distribution were determined by Logan plot analysis, using an image-derived arterial input function (IDIF) extracted from the heart blood pool of each animal, as previously described.^69, 70^

## Figures and Tables

**Table 1:** Radiochemical yields (RCY), average synthesis time and molar activity of the radiosynthesis of (*rac*)-[^18^F]OF-NB1, (*R*)-[^18^F]OF-NB1 and (*S*)-[^18^F]OF-NB1.

Cmpd	RCY (d.c.) [%]	Molar activity [GBq/pmol]	Avg. time [min]	Radiochemical purity
(*rac*)-[^18^F]OF-NB1	15 ± 5 (n = 6)	8.4 (n = 2)	97 ± 12 (n = 6)	> 99%
(*R*)-[^18^F]OF-NB1	15 (n = 2)	13.6 (n = 2)	120 (n = 2)	> 99%
(*S*)-[^18^F]OF-NB1	15 (n = 2)	10.2 (n = 2)	114 (n = 2)	> 99%

RCY: radiochemical yield; n.d.c.: non-decay corrected; d.c.: decay corrected.
